# Supplementary material for: Immunogenicity and safety of a live-attenuated SARS-CoV-2 vaccine candidate based on multiple attenuation mechanisms
Source: eLife. 2025 Feb 11;13:RP97532. doi: 10.7554/eLife.97532 (PMC11813227; doi:10.7554/eLife.97532)
Supplement: Table 1—source data 2. [file elife-97532-table1-data2.zip › Table 3- Source data 2_ORF7a-8 del PCR/Table 3-Source data 2.pdf]

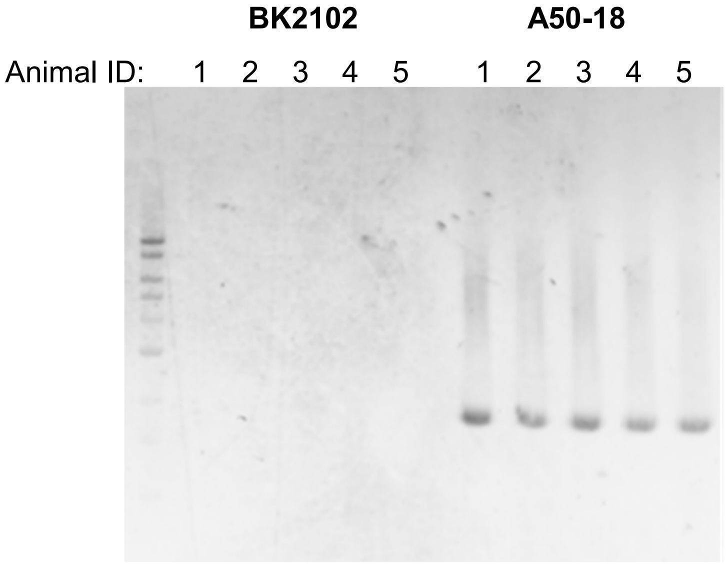


**Table 3-Source data 1.**

Table 3 was prepared based on this agarose gel electrophoresis pattern.
PCR products correspond to the amplification of the ORF7a-8 of BK2102 and A50-18 strains.
Absence of bands in BK2102 samples confirm the deletion in the vaccine candidate.
1 kb DNA Ladder was used (New England Biolabs, Inc., Cat# N3232S).
