# Supplementary figures and images for: Immunogenicity and safety of a live-attenuated SARS-CoV-2 vaccine candidate based on multiple attenuation mechanisms
Source: eLife. 2025 Feb 11;13:RP97532. doi: 10.7554/eLife.97532 (PMC11813227; doi:10.7554/eLife.97532)

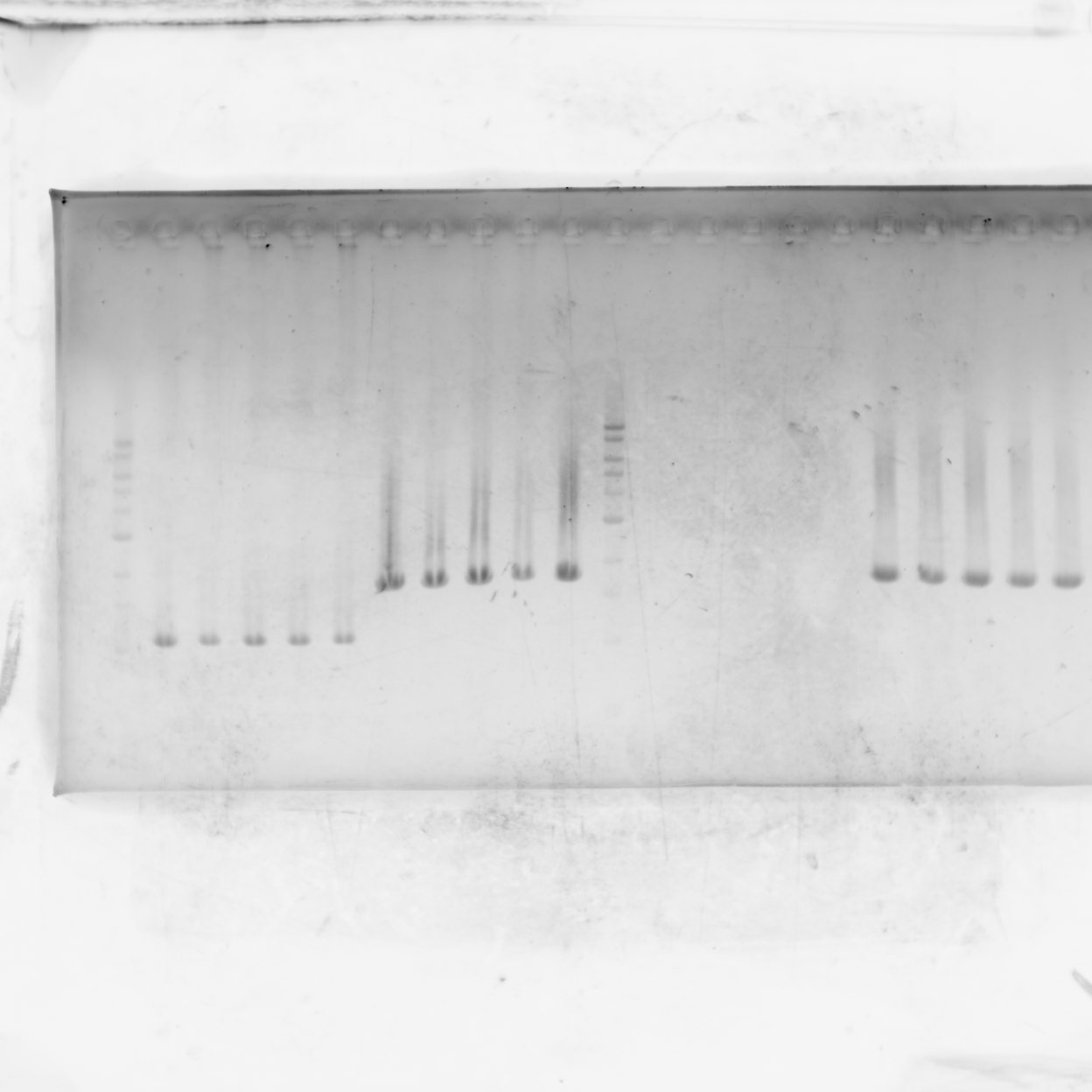

Supplement: Table 1—source data 2. [file elife-97532-table1-data2.zip › Table 3- Source data 2_ORF7a-8 del PCR/20230515_175719_Fl.tif]

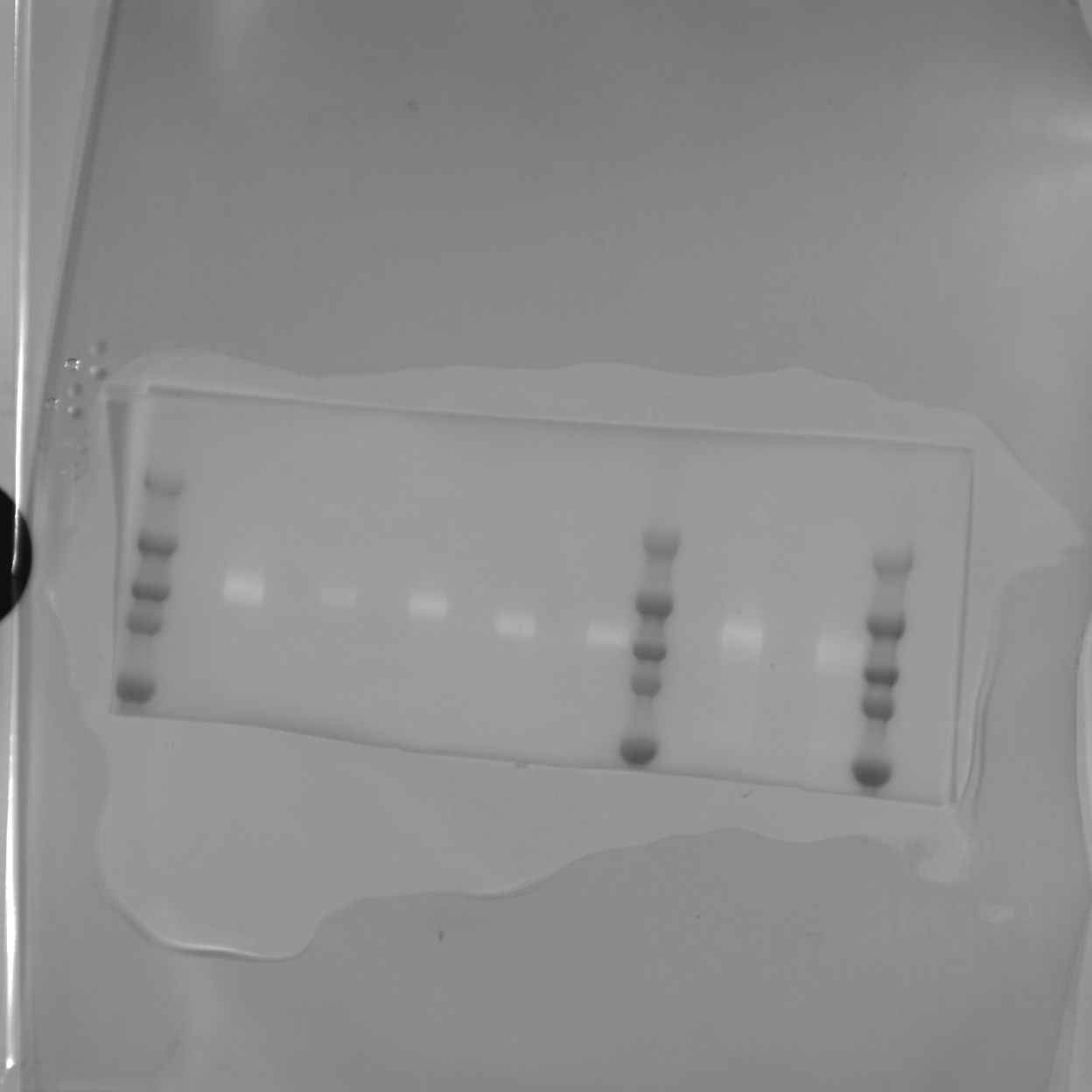

Supplement: Figure 5—figure supplement 1—source data 2. [file elife-97532-fig5-figsupp1-data2.zip › tg 20230703_180308_Ch_Marker.tif]

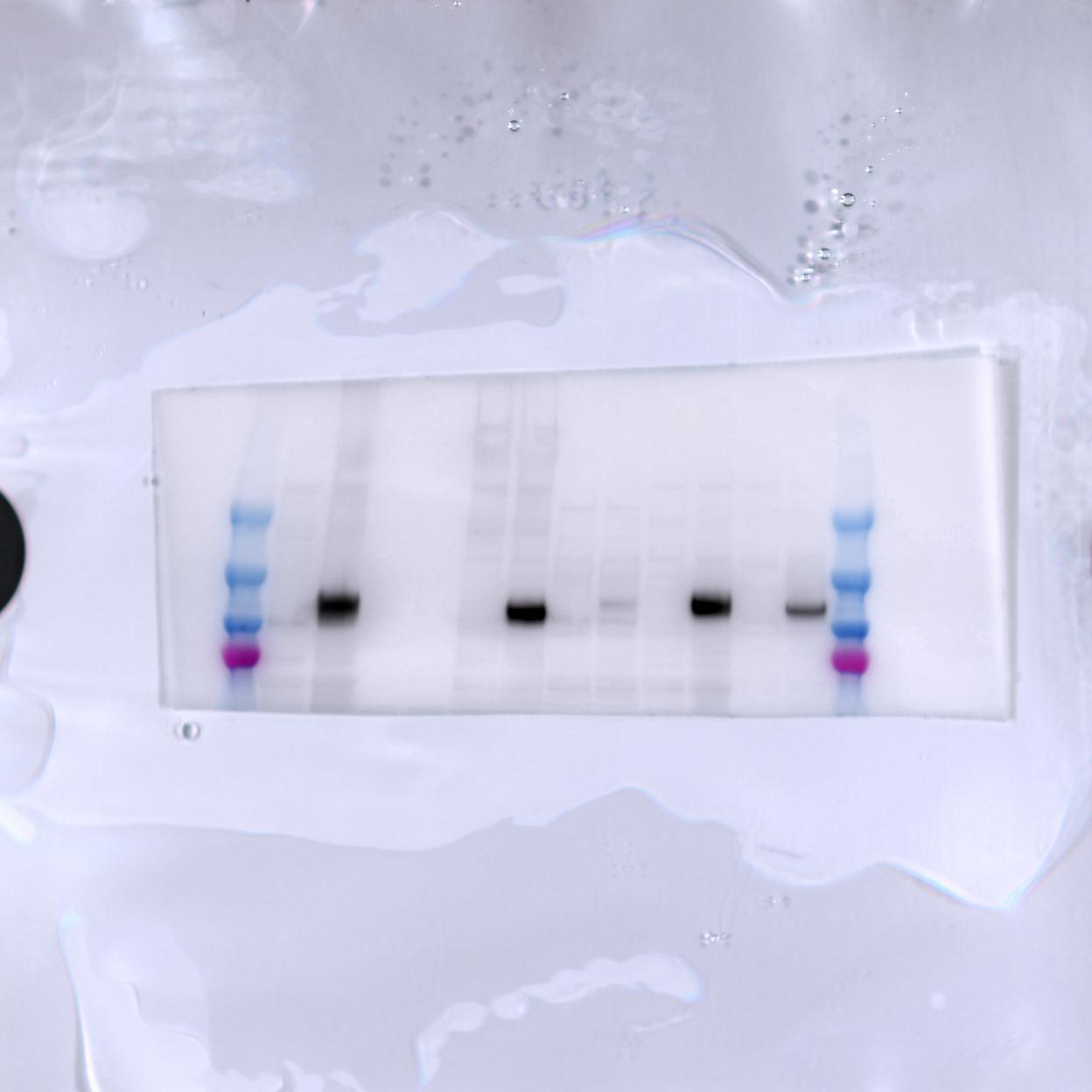

Supplement: Figure 5—figure supplement 1—source data 2. [file elife-97532-fig5-figsupp1-data2.zip › hace2 20230630_200901_Ch+Marker.jpg]

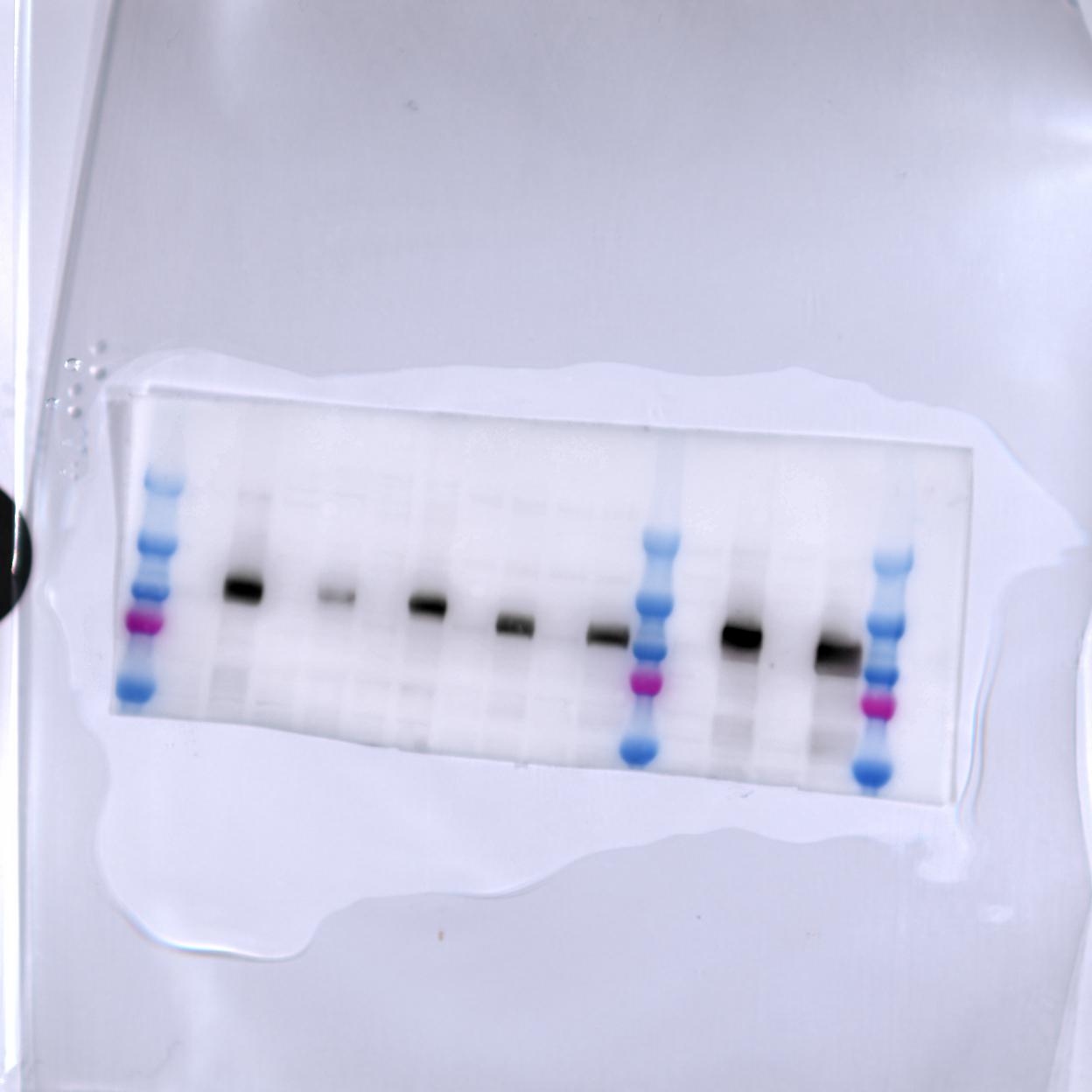

Supplement: Figure 5—figure supplement 1—source data 2. [file elife-97532-fig5-figsupp1-data2.zip › tg 20230703_180308_Ch+Marker.jpg]

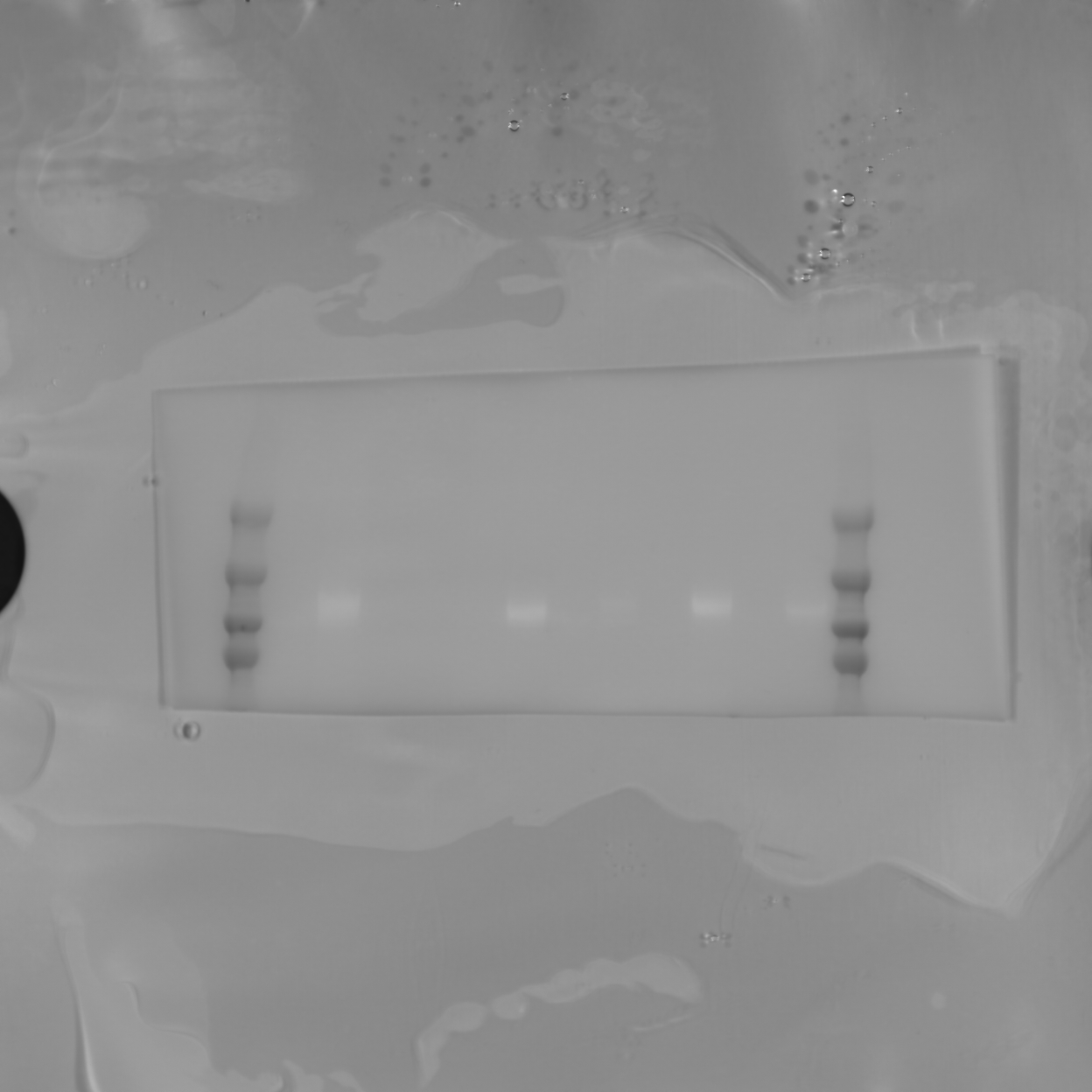

Supplement: Figure 5—figure supplement 1—source data 2. [file elife-97532-fig5-figsupp1-data2.zip › hace2 20230630_200901_Ch_Marker.tif]

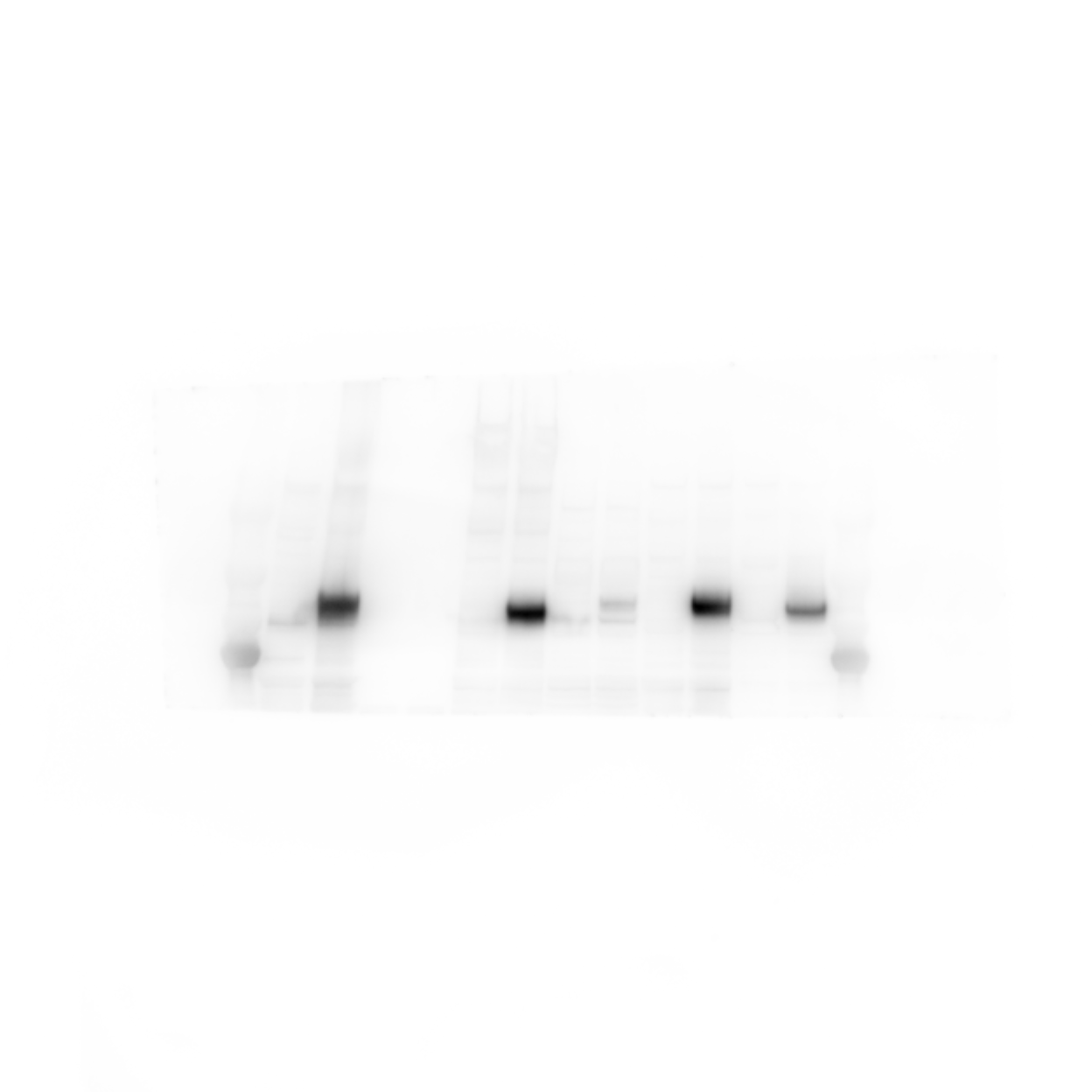

Supplement: Figure 5—figure supplement 1—source data 2. [file elife-97532-fig5-figsupp1-data2.zip › hace2 20230630_200901_Ch.tif]

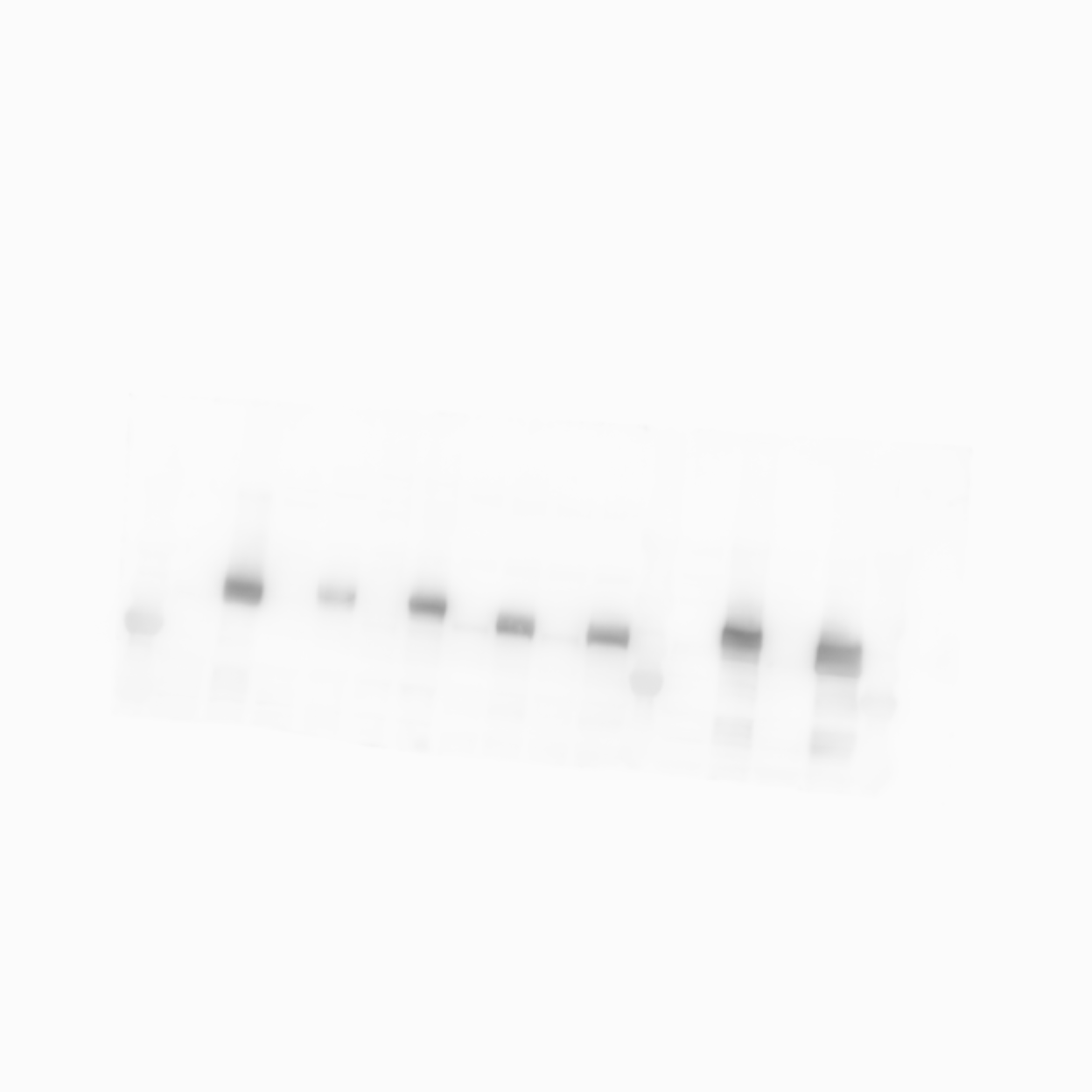

Supplement: Figure 5—figure supplement 1—source data 2. [file elife-97532-fig5-figsupp1-data2.zip › tg 20230703_180308_Ch.tif]
