# Supplementary material for: Immunogenicity and safety of a live-attenuated SARS-CoV-2 vaccine candidate based on multiple attenuation mechanisms
Source: eLife. 2025 Feb 11;13:RP97532. doi: 10.7554/eLife.97532 (PMC11813227; doi:10.7554/eLife.97532)
Supplement: Figure 5—figure supplement 1—source data 1. [file elife-97532-fig5-figsupp1-data1.zip › 141789_0_data_set_3484924_sq866q.pdf]

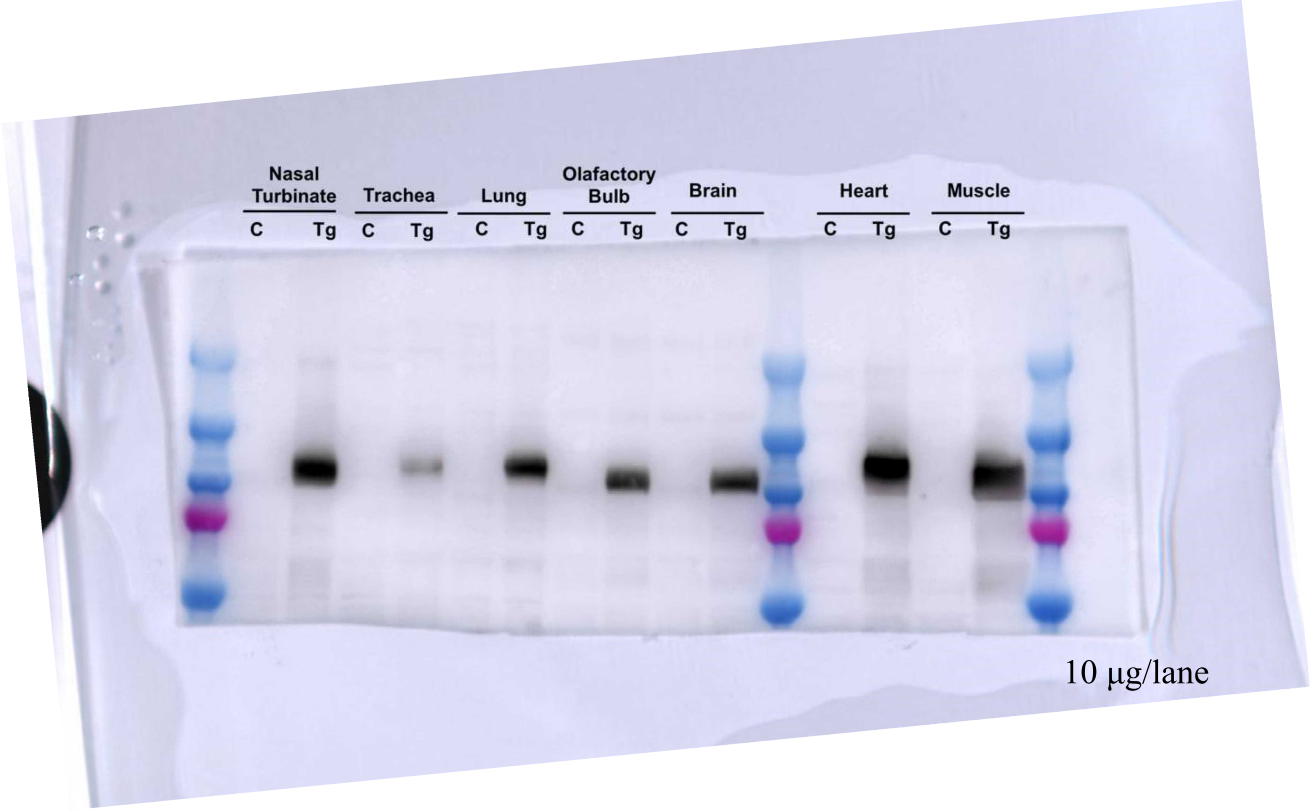

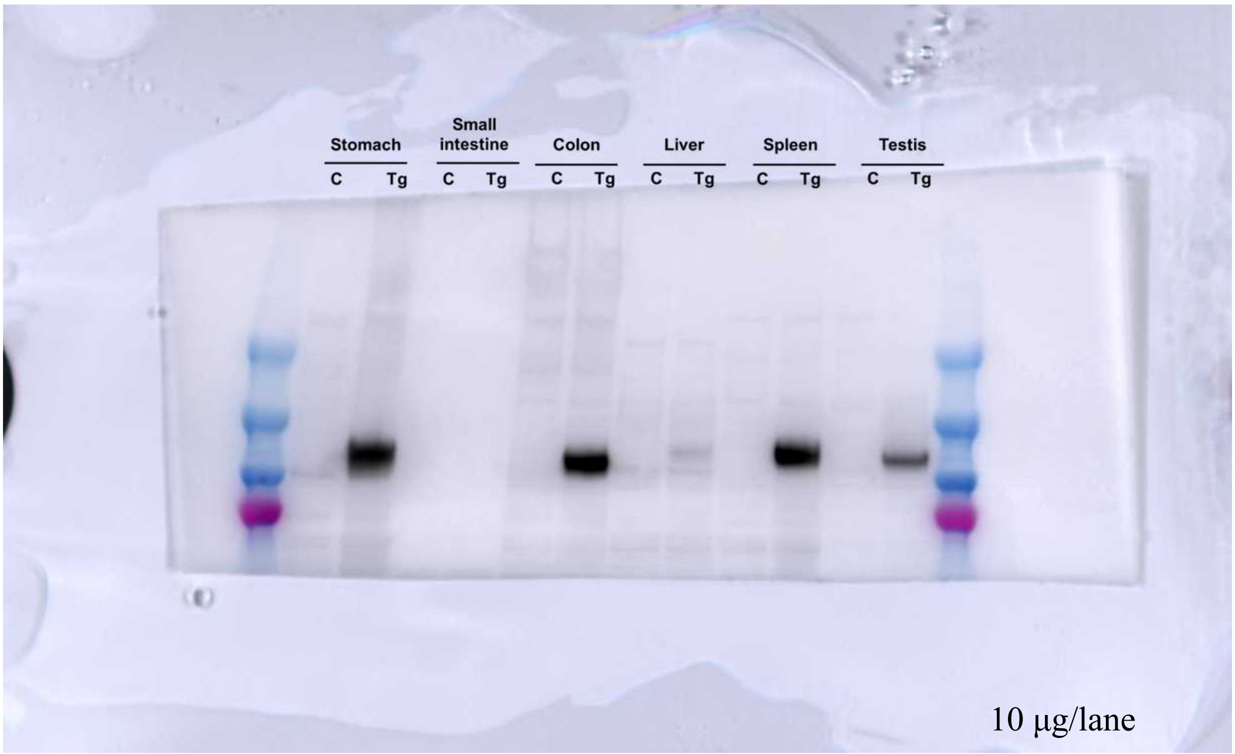

Figure 5- figure supplement 1, Source data 1. The upper membrane corresponding to Figure 5-figure supplement 1- upper panel, and the lower membrane corresponding to Figure 5- figure supplement 1- lower panel. Lane "C" showed control samples using C57BL/6N mice, and lane "Tg" showed the samples using hACE2-Tg mice. Precision Plus Protein™ Dual Color Standards were used as molecular weight markers (BIO-RAD).
